# Shattering the Amyloid Illusion: The Microbial Enigma of Alzheimer’s Disease Pathogenesis—From Gut Microbiota and Viruses to Brain Biofilms

**DOI:** 10.3390/microorganisms13010090

**Published:** 2025-01-05

**Authors:** Anna Onisiforou, Eleftheria G. Charalambous, Panos Zanos

**Affiliations:** 1 Translational Neuropharmacology Laboratory, Department of Psychology, University of Cyprus, 75 Kallipoleos Avenue, 1678 Nicosia, Cyprus; eleftheria.charalambous@med.uni-greifswald.de; 2 Center of Applied Neuroscience, 75 Kallipoleos Avenue, 1678 Nicosia, Cyprus; 3 Department of Psychiatry and Psychotherapy, University Medicine Greifswald, 1–2, Ellernholzstr., 17489 Greifswald, Germany

**Keywords:** Alzheimer’s Disease, gut–brain axis, oral microbiome, brain biofilms, viral infections, microbiome-based therapies

## Abstract

For decades, Alzheimer’s Disease (AD) research has focused on the amyloid cascade hypothesis, which identifies amyloid-beta (Aβ) as the primary driver of the disease. However, the consistent failure of Aβ-targeted therapies to demonstrate efficacy, coupled with significant safety concerns, underscores the need to rethink our approach to AD treatment. Emerging evidence points to microbial infections as environmental factors in AD pathoetiology. Although a definitive causal link remains unestablished, the collective evidence is compelling. This review explores unconventional perspectives and emerging paradigms regarding microbial involvement in AD pathogenesis, emphasizing the gut–brain axis, brain biofilms, the oral microbiome, and viral infections. Transgenic mouse models show that gut microbiota dysregulation precedes brain Aβ accumulation, emphasizing gut–brain signaling pathways. Viral infections like Herpes Simplex Virus Type 1 (HSV-1) and Severe Acute Respiratory Syndrome Coronavirus 2 (SARS-CoV-2) may lead to AD by modulating host processes like the immune system. Aβ peptide’s antimicrobial function as a response to microbial infection might inadvertently promote AD. We discuss potential microbiome-based therapies as promising strategies for managing and potentially preventing AD progression. Fecal microbiota transplantation (FMT) restores gut microbial balance, reduces Aβ accumulation, and improves cognition in preclinical models. Probiotics and prebiotics reduce neuroinflammation and Aβ plaques, while antiviral therapies targeting HSV-1 and vaccines like the shingles vaccine show potential to mitigate AD pathology. Developing effective treatments requires standardized methods to identify and measure microbial infections in AD patients, enabling personalized therapies that address individual microbial contributions to AD pathogenesis. Further research is needed to clarify the interactions between microbes and Aβ, explore bacterial and viral interplay, and understand their broader effects on host processes to translate these insights into clinical interventions.

## 1. Introduction

Alzheimer’s Disease (AD) is a progressive and chronic neurodegenerative disease (ND) affecting the central nervous system (CNS), marked primarily by memory loss and cognitive deterioration [1]. Conventional treatments offer symptomatic relief, but no pharmacotherapies have yet been able to halt or reverse its progression [2]. AD is the most common type of dementia among older adults, impacting approximately 55 million individuals globally [3]. The fundamental pathological features of AD include the development of amyloid plaques, the formation of neurofibrillary tangles, neuroinflammation, and substantial neuronal loss [1].

The amyloid cascade hypothesis, which posits that the accumulation and aggregation of amyloid-beta (Aβ) peptides in the brain are the primary drivers of AD, has long dominated the field [4]. According to this hypothesis, AD begins with the overproduction or impaired clearance of Aβ peptides, which are derived from the enzymatic cleavage of amyloid precursor protein (APP) by β-secretase and γ-secretase [4]. This leads to an imbalance between Aβ production and removal, causing the peptides to aggregate and form soluble oligomers, fibrils, and eventually extracellular amyloid plaques, a hallmark of AD [4].

Despite its prominence, the amyloid cascade hypothesis has faced significant criticism. Anti-amyloid therapies have frequently failed to deliver meaningful clinical benefits, and the hypothesis does not fully account for other pathological features of AD, such as tau hyperphosphorylation, which leads to the formation of neurofibrillary tangles, another key hallmark of AD pathology. For example, Aducanumab, an anti-amyloid monoclonal antibody approved by the Food and Drug Administration (FDA) in 2021, faced controversy over its efficacy and safety, ultimately leading to its discontinuation by its manufacturer [5]. Similarly, Gantenerumab, another amyloid-removing drug reported at the Clinical Trials on AD (CTAD) conference, reduced amyloid plaque burden but failed to slow clinical progression [6]. While the newly FDA-approved drug Lecanemab shows promise, it has been associated with significant side effects, including brain edema and intracranial bleeding [7]. Moreover, its recent recommendation for refusal by the European Medicines Agency further underscores the need for thorough investigation into its efficacy and safety. These developments raise important questions about the viability of targeting Aβ as the sole therapeutic strategy for AD and whether the amyloid cascade hypothesis adequately captures the complex and multifaceted nature of the disease.

The origins of AD are now understood to be multifactorial, arising from a complex interplay of genetic predispositions and environmental influences [8]. Traditional research primarily focuses on genetic and molecular abnormalities inherent to the disease, such as the formation of tau protein tangles and Aβ plaques. Individuals who carry one copy of the apolipoprotein E ε4 (APOE-ε4) allele have an increased risk of developing AD compared to those with the more common ε3 (APOE-ε3) allele [9,10]. The risk further increases for individuals who carry two copies of the APOE-ε4 allele [9,10]. In contrast, the APOE-ε2 allele is considered protective and is associated with a reduced risk of developing AD [9,10]. APOE-lipoproteins are essential for lipid transport, interacting with various cell-surface receptors and binding to the hydrophobic Aβ peptide, which is believed to trigger toxic processes leading to synaptic impairment and neurodegeneration in AD [9].

In addition to its role in lipid metabolism, APOE also influences immune responses, including against viral and bacterial infections, thereby modulating AD risk through its impact on immune function [11,12,13]. APOE isoforms differentially affect the immune system: APOE-ε4, the strongest genetic risk factor for AD, is associated with heightened pro-inflammatory responses, which exacerbate neuroinflammation and contribute to disease progression. Neuroinflammation is a key process in the CNS that protects the brain from pathogens, but its dysregulation contributes to the development and progression of NDs, including AD [14]. It involves activation of microglia and astrocytes, which release pro-inflammatory cytokines such as interleukin 1beta (IL-1β), interleukin 6 (IL-6), and tumor necrosis factor alpha (TNF-α), contributing to oxidative stress, impaired Aβ clearance, and neuronal damage [14].

APOE-ε4 carriers exhibit increased production of pro-inflammatory cytokines such as interleukin 8 (IL-8), IL-6, and TNF-α in response to pathogenic insults [15,16,17]. This chronic inflammatory state impairs microglial and astroglial immunomodulating functions, reducing their ability to clear Aβ and cellular debris, which promotes Aβ plaque accumulation and neuronal damage [18]. Furthermore, APOE influences the brain’s immune surveillance by modulating microglial activation and neuroinflammatory responses in the presence of Aβ plaques. APOE-ε4 is associated with more intense neuroinflammation and Aβ plaque pathology compared to APOE-ε3 and APOE-ε2, further accelerating neurodegeneration [19]. In addition to these effects, APOE-ε4 decreases plasma interleukin 7 (IL-7) levels and interleukin 7 receptor (IL-7R) signaling in in peripheral blood mononuclear cells, disrupting immune balance and enriching T cells, which may exacerbate neuroinflammation, hippocampal atrophy, and neuronal damage [20]. In contrast, APOE-ε2, the protective isoform, is linked to reduced inflammatory signaling and more effective Aβ clearance, highlighting its role in mitigating neuroinflammation and delaying AD progression [19,21]. These differences in immune modulation underscore APOE’s critical role in shaping neuroinflammatory processes and influencing the trajectory of AD pathogenesis.

APOE role in shaping the brain’s inflammatory environment and responses to microbial organisms underscores its broader contributions to AD pathogenesis. The interaction between pathogenic agents and antimicrobial properties of Aβ peptide has led to the hypothesis that Aβ accumulation in the brain may initially serve as a protective response against microbial infections or other pathogenic insults [22]. This “antimicrobial hypothesis” suggests that a pathogen could serve as a primary trigger for AD [22]. During the early stages of infection, Aβ production acts as an antimicrobial agent targeting microorganisms. However, as the infection transitions into a chronic state, sustained Aβ production results in its harmful accumulation [22]. Over time, this chronic accumulation leads to the formation of toxic aggregates and plaques, potentially creating biofilm-like structures that promote neuroinflammation and neurodegeneration [22,23]. These findings highlight the dual role of Aβ, acting as both a protective mechanism against infections and a contributor to AD pathology when dysregulated.

Emerging evidence has begun to highlight the significant role of microbial entities in the pathogenesis of AD, propelling a paradigm shift towards exploration of microbial interactions and their effects on neurodegeneration. Notably, a recent study demonstrated that fecal microbiota transplantation (FMT) from AD patients to germ-free or microbiota-depleted mice impaired hippocampal neurogenesis and cognitive performance [24]. Additionally, emerging evidence highlights the potential role of viral exposures in increasing the risk of NDs. For instance, a comprehensive study identified significant associations between 45 viral exposures and an elevated risk of NDs including AD, with some risks persisting for up to 15 years post-infection [25]. These findings suggest that a diverse pool of microbial agents may contribute to neurodegenerative processes, highlighting the importance of investigating their roles in AD pathogenesis and the mechanisms by which they exert these pathogenic effects.

The human body is an ecosystem composed of various microorganisms, including bacteria, fungi, viruses, and archaea. This community of microorganisms is referred to as microbiota that colonize different tissues of the human host such as the skin, the mouth, the brain, the vagina, the lungs, the brain, and the gastrointestinal tract (GI). Gut microbiota represent the largest reservoir of microbes, constituting the most abundant biofilm within the human body [26]. Bacterial species can grow as biofilms, which are organized communities of bacteria surrounded by a self-produced extracellular polymeric substance, composed of polysaccharides, proteins, and DNA [27]. This matrix adheres to surfaces, allowing the biofilm to establish and persist. Biofilm formation provides bacteria with enhanced resistance to antibiotics and immune system attacks, contributing to chronic infections that lead to persistent inflammation and tissue damage [27,28]. Dysbiosis in microbiota communities can have significant implications for human health, leading to the development of various health conditions, including AD.

Additionally, microbial agents, including viruses and bacteria, can influence the production and aggregation of Aβ through various mechanisms. Bacteria can produce amyloid-like proteins, such as curli from *Escherichia coli* and FapC from *Pseudomonas aeruginosa*, which can interact with host Aβ and promote its aggregation [29,30,31]. These bacterial amyloids may act as nucleation sites, accelerating Aβ fibrillization and plaque formation. Viruses, on the other hand, can modulate Aβ production and aggregation through virus–host protein–protein interactions (PPIs) [32]. For example, Herpes Simplex Virus Type 1 (HSV-1) has been shown to interact with APP, influencing its cleavage and increasing the production of Aβ [32]. Viral infections can also induce inflammatory responses, creating a microenvironment that facilitates Aβ aggregation and deposition [32,33]. These interactions suggest that microbial agents not only trigger the innate immune response involving Aβ but also directly contribute to its dysregulation, further linking infections to the progression of AD. Understanding these mechanisms is crucial for developing strategies to mitigate the microbial contribution to AD pathogenesis.

**Figure 1:**
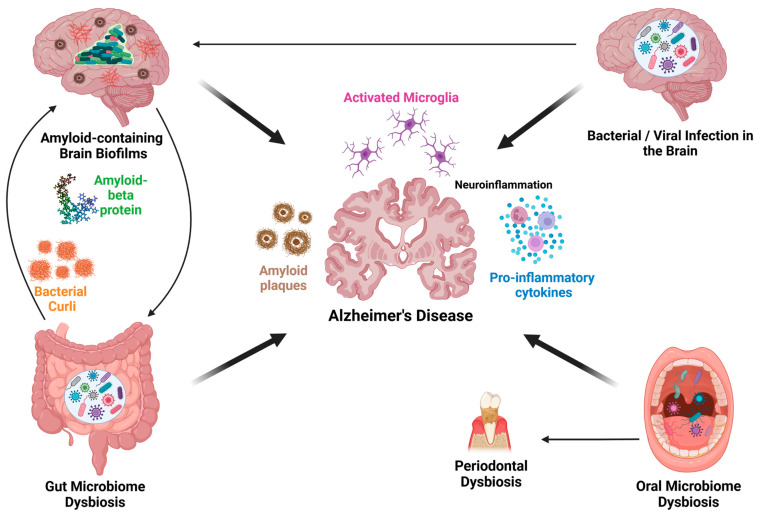
**Illustration of the multifaceted interactions between various microbial communities and AD pathogenesis.** Gut microbiome dysbiosis, with bacterial amyloids like curli produced by *Escherichia coli* and *Pseudomonas aeruginosa*, can travel to the brain via the bloodstream or vagus nerve, contributing to Aβ protein aggregation in the brain. Brain microbiota can also lead to the formation of amyloid-containing brain biofilms, further contributing to Aβ protein aggregation. Bacterial or viral infections in the brain activate microglia and trigger neuroinflammation, releasing pro-inflammatory cytokines that exacerbate AD pathology. Oral microbiome dysbiosis and periodontal disease also contribute to AD progression by promoting inflammation and possibly introducing pathogens into the brain.

This review aims to: (i) explore unconventional perspectives and emerging paradigms regarding microbial involvement in AD pathogenesis, shedding light on intricate microbe–host interactions that may facilitate neurodegeneration (Figure 1); (ii) examine how diverse microbial agents (e.g., bacteria and viruses) and microbial populations (e.g., oral, gut, and brain microbiomes), along with their interactions with the host, contribute to AD pathogenesis and how this understanding can inform future therapeutic strategies; and (iii) discuss future research directions and potential therapeutic strategies, such as microbiome-based therapies, that could benefit from these insights. By looking beyond current approaches and investigating the roles of diverse microbial communities, we can gain a more comprehensive understanding of the underlying drivers of AD pathogenesis.

## 2. Gut–Brain Axis Connection

The gut–brain axis is a well-established bidirectional communication network linking the gut microbiota and the brain, with evidence suggesting that dysbiosis in gut microbial communities can profoundly affect brain health, potentially contributing to NDs, including AD.

### 2.1. Gut Microbiota in AD: Evidence for a Causal Role in Pathogenesis

Significant evidence indicates that individuals with AD have altered microbiota composition compared to healthy controls. Although the definition of a healthy microbiome remains unclear, it is generally characterized by a balanced and diverse microbial community that supports gut health and overall well-being, with no signs of dysbiosis, chronic diseases, or related symptoms [34]. This definition is shaped by factors such as diet, lifestyle, genetics, and environmental conditions, highlighting the complexity and variability of the microbiome across individuals [34].

A recent study by Ferreiro et al. (2023) revealed significant differences in the overall composition of the gut microbiota between individuals in the early preclinical AD phase and healthy controls, even in the absence of clinical symptoms [35]. The gut microbiome composition was associated with Aβ and tau pathological biomarkers, but not with markers of neurodegeneration, suggesting that gut microbiome changes may occur during the early stages of AD [35]. Furthermore, individuals with AD exhibit decreased fecal microbial diversity and distinct microbial communities compared to healthy controls [36]. For instance, AD patients show an increased abundance of opportunistic bacteria with pro-inflammatory effects, such as *Escherichia/Shigella*, and a reduced abundance of anti-inflammatory bacteria, such as *Eubacterium rectale*, relative to healthy controls [37].

Recent findings also provide evidence for a causal role of gut microbiota in AD pathogenesis. Grabrucker et al. (2023) demonstrated that AD symptoms could be transmitted to healthy young organisms through the transfer of gut microbiota, supporting the notion that the gut microbiota play a causal role in AD pathogenesis [24]. In this study, fecal microbiota from AD patients and age-matched healthy controls were transplanted into young adult male Sprague-Dawley rats with depleted microbiota. The findings showed that transplants from AD patients into mice impaired behaviors dependent on adult hippocampal neurogenesis, with the severity of these impairments showing an inverse relationship with the cognitive scores of the donor patients, supporting the hypothesis that changes in microbiota specific to dementia donors affect cognitive function [24]. Additionally, specific changes were observed in the rats’ cecal and hippocampal metabolomes [24]. Moreover, serum from AD patients was observe to reduce neurogenesis in human cells in vitro, leading to decreased proliferation (Ki67), impaired neuronal differentiation (MAP2, DCX), and altered morphology [24]. While the serum directly impacts neurogenesis, the microbiome’s role is supported indirectly by evidence that gut microbiota composition influences plasma metabolites, which can modulate systemic effects, including neurogenesis [24]. Notably, specific microbial genera, such as *Desulfovibrio* and *Dialister*, were inversely correlated with neurogenesis markers and Mini-Mental State Examination (MMSE) scores, linking the altered microbiota in AD patients to impaired neurogenesis and cognitive decline [24]. These findings provide the first direct evidence that AD-like pathology is transmissible via gut microbiota, establishing a causal link between gut microbiota alterations and the development of AD-related neurodegenerative changes and cognitive impairments.

While data on FMT from healthy human individuals to AD transgenic mouse models are limited, studies using animal models have demonstrated the therapeutic potential of microbiota transfer. For example, FMT from healthy wild-type donor mice to AD transgenic mouse models has been shown to reduce pathology and improve cognitive function [38,39,40].

### 2.2. Mechanisms of Microbiota Influence in AD

Gut microbiota are crucial for maintaining homeostasis within the human body, playing a pivotal role in regulating various physiological functions, including metabolic processes, endocrine signaling, and immune responses [41]. These microorganisms facilitate human metabolism by producing enzymes absent in the human genome, enabling the breakdown of complex carbohydrates, particularly plant-derived polysaccharides such as cellulose and resistant starch, into simpler forms that can be absorbed and utilized by the host [42,43]. Additionally, gut microbiota contribute to the synthesis of vital nutrients, such as vitamin K and B vitamins, which are crucial for metabolic and physiological functions [42,43].

One of the most significant contributions of gut microbiota is the production of short-chain fatty acids (SCFAs) like acetate, propionate, and butyrate. These metabolites are primarily formed in the colon through the fermentation of dietary fiber and resistant starch by gut bacteria [44]. SCFAs regulate host metabolism, modulate immune function, and influence neuro-immunoendocrine processes, underscoring their importance in maintaining overall health [45]. Additionally, microbiota are also involved in the production of neurotransmitters (NTs), such as serotonin, dopamine, glutamate, tryptophan, and gamma-aminobutyric acid (GABA), influencing mood, cognition, and behavior [46,47]. Disruptions in the synthesis of these neurotransmitters have been linked NDs, including AD [48,49,50].

Although the precise mechanisms linking gut microbiota dysbiosis to the pathogenesis of AD via disrupted gut–brain communication remain unclear, several potential pathways have been identified through which gut microbiota can influence brain function. These include altering blood–brain barrier (BBB) permeability [51]; modulating immune system processes and immunological memory formation through microbiota-derived metabolites [52], directly affecting the vagus nerve [53]; and producing neuroactive metabolites such as SCFAs and NTs [45,52]. Additionally, gut bacteria can produce amyloid-like proteins that share structural similarities with human Aβ amyloids, potentially influencing amyloid aggregation and pathology [29,30,31].

### 2.3. Blood–Brain Barrier Permeability

The ability of the gut microbiota to increase BBB permeability can allow harmful substances to penetrate the brain, triggering neuroinflammation. This inflammatory response can promote the formation of Aβ plaques and neurofibrillary tangles [54]. The BBB regulates the exchange of molecules and nutrients between the bloodstream and brain tissue, ensuring CNS homeostasis. Studies show that germ-free mice, starting from the prenatal stage, exhibit increased BBB permeability compared to pathogen-free mice with normal gut microbiota flora [51]. This increased permeability persists after birth and into adulthood and is associated with reduced levels of tight junction proteins, such as occludin and claudin-5, which are essential for maintaining endothelial barrier integrity [51]. Remarkably, introducing a pathogen-free gut microbiota into germ-free adult mice reduces BBB permeability and restores tight junction protein expression [51]. These findings suggest that the interaction between gut microbiota and the BBB begins in utero and continues to play a vital role in maintaining BBB integrity throughout life.

Although the exact mechanisms by which gut microbiota regulate the BBB remain unclear, it is hypothesized that microbiota-derived SCFAs play a vital role in maintaining BBB integrity under various pathological conditions, including AD [55]. In hypercholesterolemic APOE-knockout (ApoE−/−) rats, a model frequently used to investigate lipid-associated disorders and neurodegenerative processes such as AD, monobutyrin, and monovalerin, esters of microbiota-derived SCFAs, was administered to evaluate their potential to counteract the detrimental effects of high-fat intake and APOE deficiency [56]. These factors are known to impair gut–brain barrier function and contribute to obesity and neuropathological disorders, including AD [56]. The study demonstrated that monobutyrin and monovalerin significantly increased the expression of critical tight junction proteins, including occludin and zonula occludens-1 (ZO-1), in the brains of high-fat-fed ApoE−/− rats [56]. These proteins are key components of the tight junction complex that regulates paracellular permeability and ensures barrier function. By enhancing the expression of these proteins, monobutyrin and monovalerin may help restore or maintain BBB integrity, potentially counteracting the detrimental effects of high-fat diets and genetic risk factors on barrier dysfunction.

### 2.4. Microbiota-Derived Metabolites and Neurotransmitters: Diverse Effects on AD Processes

Additionally, the gut microbiota can influence neurotransmitter production, contributing to cognitive decline and behavioral symptoms [57]. In APOE-knockout (ApoE−/−) rats, supplementation with monobutyrin and monovalerin increased levels of the neurotransmitter GABA in the brain [58]. GABA plays a critical role in mitigating excitotoxicity and neuroinflammation, two hallmark processes in AD pathogenesis, suggesting that modulating microbiota may have direct neuroprotective effects. Monobutyrin supplementation also increased the abundance of *Adlercreutzia* in the cecum [58], a bacterial genus identified in genome-wide association studies as a protective factor against AD [59]. Conversely, monovalerin supplementation reduced levels of *Proteobacteria*, a bacterial group frequently linked to gut dysbiosis and systemic inflammation [58]. Notably, AD patients exhibit elevated levels of *Proteobacteria* compared to healthy controls, further implicating dysbiosis in disease progression [36]. These findings highlight how the gut microbiota, through their influence on neurotransmitter regulation, can contribute to the pathogenesis of AD.

Gut bacteria can also influence the production of serotonin by metabolizing dietary tryptophan via the kynurenine pathway, which has been linked to cognitive and mood changes in AD [60]. Decreased serotonin levels has been observed in the brain and cerebrospinal fluid of AD patients, as well as a reduction in serotonergic receptors in various brain regions, including the amygdala and hippocampus [61,62]. Selective serotonin reuptake inhibitors (SSRIs) improve behavioral and cognitive symptoms in AD patients [63,64]. Dysfunction of the serotonergic system in AD has been associated with the development of behavioral and psychological symptoms of dementia (BPSD), which are frequently observed in AD patients [65]. Notably, serotonin conjugated with isoalantolactone inhibited β-secretase enzyme (BACE-1), prevented Aβ1–42 aggregation, and demonstrated neuroprotective effects in SH-SY5Y neuroblastoma cells, while enhancing learning and memory in the 5xFAD mouse model of AD [66].

Microbiome-derived NTs can also regulate microglial activity, impacting CNS responses [67] and potentially affecting microglial homeostasis in the brain. Microglia, which are critical to AD development, play a key role in engulfing and clearing apoptotic neurons, bacteria, lipoproteins, and Aβ [68]. Through the production of NTs, gut microbiota can modulate microglial function, altering their phagocytic activity and inflammatory responses. Additionally, NTs act as neuromodulators, influencing both innate and adaptive immune responses, as leukocytes express receptors for NTs such as glutamate, dopamine, serotonin, and acetylcholine [69,70]. The interaction between the brain and the peripheral immune system is bidirectional, with leukocytes synthesizing and releasing NTs as well as producing cytokines essential to neuroimmunomodulatory pathways [70]. During infections, immune mediators signal the CNS to amplify immune responses via hormonal and neural mechanisms. Once the infection is resolved, the CNS downregulates immune activity to restore homeostasis [71]. Thus, modulation of NTs by gut microbiota not only directly impacts the progression of NDs, but also indirectly influences neuroinflammation by altering NT production and immune regulation.

A study using APPswe/PSEN1dE9 transgenic male mice showed that a reduction in microbiota diversity was accompanied by decreased levels of SCFAs such as acetate, propionate, and butyrate, typically produced by commensal bacteria by breaking down dietary fiber [72]. The typical production ratios of these SCFAs are 60:20:20, respectively, and are directly affected by dietary fiber content [72]. In this model, reduced SCFA levels were associated with alterations in over 30 metabolic pathways potentially linked to amyloid deposition, suggesting a direct connection between gut microbial changes and AD pathology [72]. FMT from healthy mice into the ${ADLP}^{APT}$ transgenic mouse model of AD resulted in a reduction in Aβ plaque and neurofibrillary tangle formation, as well as an improvement in cognitive deficits [39].

SCFAs and other microbiota-derived metabolites, including NTs produced by gut microbes, play crucial roles in maintaining gut integrity, modulating immune responses, and influencing neural function [41,54,57,73,74,75,76]. These metabolites can cross the BBB and interact with neural pathways, potentially inducing neuroinflammation and neurodegeneration, as well as impairing cognitive functions [41,54,57,73,74,75,76].

Gut microbiota actively modulate host metabolic processes, including lipid metabolism, by transforming and synthesizing lipids through both diet-dependent and diet-independent pathways [77]. Lipidomic and metabolomic studies have consistently identified altered lipid levels in the brains of early-stage AD patients [78]. These alterations are linked to various pathological mechanisms in AD, such as amyloidogenesis, bioenergetic deficits, oxidative stress, neuroinflammation, and myelin degeneration [78]. Lipids, which make up about 50% of the brain’s mass, including fatty acids, cholesterol, phospholipids, and sphingolipids, play a crucial role in proper brain function [79]. A multi-omics study revealed a potential link between gut microbiota and host glycerophospholipid metabolism to be linked to neuroinflammation levels in APP/PS1 mice, a transgenic mouse model of AD [80].

### 2.5. Immunomodulatory Effects of Gut Microbiota

A bidirectional crosstalk exists between gut bacteria and the immune system, with perturbations in microbiome-immune system interactions hypothesized to mediate NDs, like AD, via neuroinflammation [81,82]. The interaction between gut microbiota and the immune system is a highly regulated and dynamic process. The immune system maintains gut homeostasis by reacting to pathogenic organisms such as viruses and tolerating beneficial microbiota [83,84]. Conversely, gut commensal bacteria can regulate immune responses, influencing hematopoiesis and shaping intestinal immune cell populations [85]. Gut microbiota can also influence AD pathogenesis by affecting innate and adaptive memory formation through microbiota-derived bioactive molecules like SCFAs and NTs [86,87]. These interactions can lead to heightened immune responses and chronic inflammation, which contribute to neurodegeneration. Although several mechanisms have been identified, the exact ways in which gut bacteria influence immune memory formation remain an emerging area of investigation [86]. Innate immune memory, a relatively new concept, shows that cells involved in the innate immune response can exhibit heightened responses to past insults or heterologous stimuli, similar to adaptive immune responses [88,89,90]. Thus, innate immune memory can also potentially contribute to AD neuroinflammation.

In the APP23 transgenic mouse model of AD, it was demonstrated that peripheral stimuli could train microglia cells in the brain to develop innate immune memory, which in turn exacerbated β-amyloidosis [91]. On the other hand, inducing innate immune tolerance in these microglia alleviated this effect [91]. Microglia, which serve as the resident macrophages of the CNS, possess the ability to be “primed” by their prior exposure to inflammatory stimuli, allowing them to develop innate immune memory [92]. Based on the nature of the initial stimulus, subsequent exposure to inflammatory signals may either amplify their response or lead to a diminished reaction [92]. These findings indicate that microglia may undergo a form of classical conditioning based on initial stimuli, influencing their behavior upon re-exposure and thereby impacting the progression of AD.

This evidence indicates that microbiota may play a significant role in AD through their impact on the immune system, particularly in modulating microglia in the brain. The ability of microglia to develop innate immune memory and respond differently to inflammatory stimuli suggests that gut microbiota could influence the progression of AD by modulating immune responses. Through a multi-dimensional, knowledge-driven systems approach to explore the interactions between microbial metabolites, microglia, and AD, it was found that SCFAs were prominently ranked among the microbial metabolites linked to abnormal microglia activity in AD [93].

### 2.6. Gut-Derived Aβ and Microbial Amyloids

AD pathophysiology has traditionally been viewed to originate from the brain, but emerging evidence from AD patients and animal models indicates that Aβ peptide overexpression also occurs in the GI tract [94,95,96]. In the APP/PS1 transgenic AD mouse model, young mice exhibited enteric Aβ accumulation, gastrointestinal dysfunction, and inflammation in the colon [95], suggesting that the enteric nervous system (ENS) also undergoes neuropathological changes similar to those observed in AD brains. These intestinal alternations may serve as early indicators contributing to the development of brain pathology [95]. Additionally, gut levels of Aβ42 have been shown to increase with age in both wild-type and APP/PS1 mice [94].

FMT from aged APP/PS1 mice into wild-type mice resulted in elevated gut BACE1 and Aβ42 levels, which triggered neuroinflammation, a key feature of early AD pathology, suggesting that gut microbiota regulate the production of Aβ in the gut [94]. Importantly, Aβ42 originating from the gut was primarily transported to the brain through the bloodstream rather than through the vagal nerve [94]. However, another study, conducted by injecting Aβ into the GI tract of ICR mice, showed that enteric Aβ oligomers induced amyloidosis in the CNS and AD-like dementia through vagal pathways [96]. These studies present seemingly contradictory evidence regarding the transport mechanisms of gut-derived Aβ to the brain: one study suggests the bloodstream as the primary route, while the other implicates vagal nerve involvement. This discrepancy may be due to differences in experimental models, forms of Aβ studied, temporal dynamics, detection techniques, and the complex interplay within the gut–brain axis, highlighting the need for further research to clarify these mechanisms. Additionally, in a Tg2576 mouse model of AD, dysregulation of gut homeostasis was observed prior to the accumulation of Aβ in the brain [97], suggesting that gut dysfunction may precede and contribute to brain neuropathology.

Overall, these findings highlight the importance of the gut–brain axis in AD pathology and suggest that the initiation of AD might originate from outside the brain, specifically in the GI tract and subsequently translocating to the brain via the bloodstream or vagus nerve [94,96]. This highlights the complexity of Aβ transport mechanisms and suggests that multiple pathways may be involved under different circumstances, reinforcing the importance of gut–brain interactions in the development and progression of AD.

Interestingly, opportunistic pathogens from the gut such as *Pseudomonas aeruginosa* and *Escherichia coli* can produce specific proteins, i.e., FapC and CsgA, respectively, that can self-assemble into fibrillar structures [29,30,31]. These bacterial amyloids form biofilms and are analogous to human Aβ amyloids [29,30,31]. A study on Tg2576 AD mice revealed a significant increase in bacterial amyloid curli and toll-like receptor 2 (TLR2) mRNA levels in the gut, linked to elevated colonization of gram-positive bacteria in the ileum [98]. Vagus nerve activation by bacterial curli indicated a gut–brain signaling pathway. Elevated TLR2 and PGP9.5 levels in the gut epithelium suggested a connection between bacterial amyloids and neuroendocrine activation [98], highlighting a potential mechanism through which gut microbiota could influence AD pathology. A recent study has demonstrated that Aβ monomers have the ability to target and break down microbial amyloids, specifically FapC and CsgA, indicating a possible involvement of Aβ in the gut–brain connection [99]. It was also demonstrated that once these microbial fibrils are remodeled by Aβ, they lose their cell adhesion properties and are detoxified [99]. This indicates that Aβ monomers may have an anti-biofilm function, which could have implications for the development of future therapies targeting both AD and infections [99]. These findings suggest that gut microbiota can produce bacterial amyloids, which mimic human amyloids and are targeted by Aβ monomers. These monomers can remodel and detoxify microbial amyloids, reducing their biofilm-forming and adhesive properties, highlighting a potential protective role for Aβ in the gut–brain axis. This anti-biofilm function may represent an evolutionary adaptation to combat microbial threats, but also underscores the dual role of Aβ in both defense and disease.

## 3. Beyond the Gut: The Oral Microbiome’s Role in AD

While the gut microbiome has garnered significant attention in AD research, dysbiosis from microbial communities from other body sites, such as the oral cavity, may also contribute to the development and/or progression of AD. Recent studies have suggested a link between periodontal disease, changes in the oral microbiome, and the onset of cognitive decline and AD [100,101].

The oral cavity is as a complex microbial ecosystem hosting diverse microorganisms, including bacterial, fungal, and viral communities. Its barrier function is maintained by the oral mucosa, saliva, and the immune system. The oral mucosa provides a physical barrier, while saliva contains antimicrobial proteins, enzymes, and immunoglobulins (e.g., IgA) that regulate microbial growth and maintain homeostasis [102]. The oral mucosa, which forms the lining of the oral cavity, acts as a vital entry point to the body and serves as the first line of defense against invading pathogens [103].

In response to certain triggers, such as poor oral hygiene, dietary factors, systemic health conditions, smoking, alcohol use, stress, medication use, age-related changes, or viral/bacterial infections and injuries, the oral microbial community can shift [104]. This can lead to an imbalance in the oral microbiome, resulting in an increased abundance of opportunistic bacteria such as *Porphyromonas gingivalis*, *Fusobacterium*, and *Prevotella* [105]. This dysbiosis triggers host inflammatory responses, which can exacerbate oral diseases such as periodontitis or gingivitis [105,106,107,108]. Interestingly, the concept of “leaky gut” syndrome, characterized by compromised intestinal barrier integrity, can also be applied to the oral cavity. Conditions like periodontitis and gingivitis are associated with a “leaky gum” phenomenon [109], where disruptions in the oral barrier may lead to systemic inflammation and neuroinflammation, as well as potentially contribute to neurodegenerative processes, including those observed in AD.

### 3.1. The Oral Microbiome and AD Pathogenesis

*P. gingivalis*, primarily known as a key pathogen associated with chronic periodontitis in the oral cavity, has also been detected in other parts of the body. Notably, it has been found in the brains of AD patients along with its toxic proteases, known as gingipains, which have been correlated with tau pathology [100]. Gingipains can cleave tau proteins and arginine residues on APOE [110,111], thus contributing to the formation of neurofibrillary tangles and amyloid plaques. Inhibiting gingipains in female BALB/c mice, following the injection of gingipains into the hippocampus, has been shown to reduce *P. gingivalis* brain infection, block Aβ production, decrease neuroinflammation, and protect neurons, suggesting that gingipain inhibitors could be valuable in treating AD [100]. Additional observational studies further support this hypothesis by providing evidence of a connection between oral bacteria, particularly *P. gingivalis,* and AD [112,113,114].

Findings from ApoE−/− transgenic mouse models of AD demonstrated that the oral pathogen *P. gingivalis* can invade the brains of these mice, with the incidence of invasion increasing with the duration of infection [115]. Once in the brain, *P. gingivalis* activates the complement system, specifically C3 and C9, leading to opsonization in hippocampal neurons and associated neuronal damage, which likely contributes to the neuroinflammation observed in AD [115]. Moreover, virulence factors produced by oral bacterial pathogens, such as gingipains, play a critical role in compromising the gum endothelial barrier’s permeability [116]. This “leaky” barrier allows pathogens and their toxic components, like gingipains, to enter the bloodstream, potentially reaching the brain and triggering neuroinflammation and neuronal damage.

Additionally, a study using positron emission tomography imaging demonstrated that periodontal disease, measured by clinical attachment loss, is associated with an increased Aβ load in the brains of elderly individuals with normal cognitive function [101]. This findings provided the first evidence in humans linking periodontal disease to brain Aβ accumulation, supporting earlier animal studies that peripheral inflammation and infections can promote Aβ build-up in the brain independently of cognitive impairment [101]. Furthermore, the enhanced detection of anti-IgG associated with periodontal clusters has been linked to an increased risk of AD-related mortality [116,117]. These findings underscore the oral–brain axis as a key pathway in AD progression, emphasizing the need to consider periodontal disease and oral microbiota as modifiable risk factors in preventing or managing AD. The oral–brain connection also highlights the potential for developing novel therapies targeting oral pathogens and their systemic effects to mitigate brain inflammation and Aβ accumulation.

Alcohol, particularly excessive consumption and binge drinking is a significant confounding factor in microbiome studies and has been shown to negatively impact the oral microbiome. Binge drinking induces shifts in the oral microbial community, promoting the growth of harmful bacteria that can trigger systemic inflammation [118,119,120]. This inflammation increases BBB permeability, allowing pro-inflammatory cytokines to cross into the brain and contribute to neuroinflammation, a hallmark of AD [118,119,120]. Meta-analyses and pathway analyses suggest that elevated ethanol levels in binge drinkers alter the oral microbiome and may contribute to AD pathogenesis through activation of eIF2, regulation of eIF4, p70S6K, and mTOR signaling pathways [118].

### 3.2. The Oral-Gut–Brain Axis: A Holistic Microbiome Perspective

The interorgan microbial network is increasingly recognized as a crucial factor in regulating both physiological functions and disease processes [121]. The oral cavity and gut, as the two largest microbial ecosystems, are typically distinct due to the oral–gut barrier [121]. However, unlike other barriers such as the gut barrier and BBB, the oral–gut barrier is relatively less understood and lacks a clear physical separation. Instead, it is characterized by dynamic microbial, immunological, and chemical interactions that influence both local and systemic health.

This barrier functions as a regulatory interface between the oral and gut microbiomes, mediated by the continuous passage of saliva, oral bacteria, and immune signals. When the oral–gut barrier is compromised, oral bacteria can migrate to the intestinal mucosa either through the digestive tract via swallowing or through the bloodstream [121]. This microbial movement between the oral and gut environments supports the concept of an oral–gut microbiome axis, which plays a significant role in disease pathogenesis [121].

Disruptions in the oral–gut barrier, such as those caused by poor oral hygiene, dietary imbalances, or systemic conditions, can lead to changes in both oral and gut microbial compositions. These changes can result in an imbalance of pro-inflammatory and anti-inflammatory microbial metabolites, contributing to systemic inflammation and neuroinflammation [122], both of which are implicated in AD. For example, certain oral pathogens like *P. gingivalis* can induce local inflammation in the mouth and, through microbial migration, impact distant organs such as the gut and brain via the oral–gut–brain axis [120]. When oral pathogens colonize the gut, they can trigger or exacerbate gut dysbiosis, leading to increased intestinal permeability (commonly referred to as “leaky gut”) [120]. This allows bacterial components, such as lipopolysaccharides (LPS) and other toxins, to enter the bloodstream, where they can reach the brain [120]. Once in the brain, these components can trigger neuroinflammation and contribute to the formation of Aβ plaques [120].

Understanding the interactions between the oral and gut microbiota, as well as their collective influence on the brain through the oral–gut–brain axis, is essential for advancing therapeutic strategies to prevent or mitigate AD. A holistic approach to microbiome research that considers the interconnectedness of microbial communities and their impact on systemic and neurological health is critical for addressing the complexities of AD pathogenesis. This includes exploring novel interventions targeting the oral–gut barrier to restore balance and reduce inflammation, potentially offering new avenues for AD prevention and treatment.

## 4. The Amyloid–Brain Biofilm Hypothesis

The brain, once thought to be a completely sterile environment, is now recognized as a site where microorganisms can reside. Recent studies have revealed a persistent presence of microbes in both healthy and diseased human brains [123,124]. For instance, significant microbial diversity has been identified in the human brain, including bacteria, fungi, and viruses. Specific microbes, such as *Streptococcus*, *Staphylococcus*, *Sphingomonas*, *Aspergillus*, *Candida*, and *Cryptococcus*, are found in higher numbers in the brains of individuals with AD compared to healthy control subjects [125]. However, not all AD samples have tested positive for these microbes, which may reflect the localized nature of microbial contributions to AD, as certain brain regions affected by infection might not have been analyzed in every case [125].

Further evidence implicates bacterial components in AD pathology. For example, Gram-negative bacterial molecules, including *Escherichia coli* K99 pili protein and LPS, have been found at elevated levels in the brains of individuals with AD compared to healthy controls [126]. LPS has been shown to colocalize with Aβ in plaques and around blood vessels in AD brains [126]. These findings suggest that bacterial infections and their components, such as LPS, may play a significant role in the progression of AD neuropathology [126]. Other studies have also consistently demonstrated the detection of bacteria and fungi in the brains of individuals with AD, further supporting the hypothesis of microbial involvement in the disease [124,127].

One intriguing aspect of these findings is the potential role of microbial infections in promoting the formation of amyloid-containing biofilms in the brain, representing a novel dimension of AD pathogenesis. Bacteria such as *Pseudomonas aeruginosa* and *Escherichia coli* produce bacterial amyloids that form biofilms, which are structurally and functionally analogous to human Aβ amyloids [29,30,31]. This evidence suggests that the accumulation of Aβ in the brain may, in part, be driven directly by microbial agents themselves, highlighting the possibility that microbial amyloids contribute to both the formation and persistence of amyloid plaques in AD.

## 5. The Role of Viruses in AD Pathogenesis

### 5.1. Viruses as Environmental Factors in AD

Viruses, alongside bacteria, are essential components of the human microbiome, collectively referred to as the virome. As obligate intracellular parasites, viruses depend on host cellular machinery for survival and replication, hijacking host processes to meet their needs [128,129]. Over time, they have developed sophisticated mechanisms to manipulate host systems, including immune evasion strategies such as disrupting antigen presentation and mimicking host immune processes to avoid detection [130]. These evolutionary adaptations enable viruses to modulate host pathways via virus–host PPIs, influencing disease-associated mechanisms and potentially contributing to NDs, including AD [33,131].

The viral hypothesis of AD suggests that viral infections are critical environmental factors that may accelerate the development and progression of AD [132]. Studies have shown that individuals infected with HSV-1 are at an increased risk of AD, with post mortem analyses revealing higher HSV-1 levels in the brains of AD patients [133,134,135]. This observation supports the “antimicrobial protection hypothesis”, which proposes that increased microbial presence in the brain triggers Aβ deposition as an innate immune response to combat pathogens [22]. While initially protective, this response sustains neuroinflammation and promotes neurodegeneration [22].

Epstein–Barr Virus (EBV) has also been implicated in AD. EBV was detected in 6% of brain samples from AD patients, with a notable association between EBV-positive samples and individuals carrying the APOE-ε4 allele [136]. In peripheral blood leukocytes (PBL), EBV was present in 45% of AD patients compared to 31% of controls, indicating a strong link between EBV presence and an increased risk of AD [136]. A five-year follow-up study revealed that individuals who developed AD showed rising EBV positivity in their PBL and elevated immunoglobulin G (IgG) antibody levels, further suggesting a connection between EBV persistence and AD onset [136]. Additionally, sex- and region-specific transcriptomic analysis of post mortem brain samples revealed that the EBV pathway was among the top pathways in female AD patients compared to female controls [137]. This pathway was specific to the CA1 region of the hippocampus when compared to males with AD, suggesting a female-specific vulnerability to EBV-related immune responses and region-specific susceptibility within the CA1 but not the CA3 subfield [137].

Other viruses associated with AD, include Hepatitis C Virus (HCV), Human Herpesvirus 6A/B (HHV-6A/B), Human Cytomegalovirus (HCMV), Varicella–Zoster Virus (VZV), Human Herpesvirus 7 (HHV-7), Severe Acute Respiratory Syndrome Coronavirus 2 (SARS-CoV-2), and Herpes Simplex Virus Type 2 (HSV-2) [32,133,136,138,139,140,141,142]. Notably, SARS-CoV-2, responsible for the COVID-19 pandemic, has been linked to AD-like pathologies such as memory impairment and brain fog, particularly in patients with post-COVID-19 cognitive symptoms [143,144,145]. An analysis of nearly 450,000 electronic health records revealed that multiple viral infections are associated with a higher risk of developing NDs, including AD [25]. However, the precise pathological mechanisms by which viruses can directly or indirectly contribute to the emergence of AD remain unclear.

### 5.2. Virus–Host Interactions and Amyloid Formation

Research using system bioinformatics methodologies suggests a heightened risk of developing AD following SARS-CoV-2 infection [33]. This risk is potentially mediated by the virus’s influence on biological processes associated with the disease through virus–host PPIs [33]. Furthermore, SARS-CoV-2 may induce AD through autoreactivity, where molecular mimicry between SARS-CoV-2 epitopes and host proteins associated with neurodegeneration, such as APP and microtubule-associated protein tau (MAPT), might occur [33]. This connection could explain the observed approximately 17% increase in Alzheimer’s-related deaths in 2020, coinciding with the pandemic [146]. However, further research is required to absolutely confirm a direct causal relationship between the two.

Eimer et al. investigated whether viral infections, specifically members of the *Herpesviridae* family, could seed and accelerate Aβ deposition [147]. Injecting HSV-1 into the brains of young 5xFAD mice, which overexpress amyloid, and wild-type mice led to a rapid increase in Aβ deposition [147]. Interestingly, the transgenic 5xFAD mice survived longer than controls when exposed to a lethal dose of HSV-1 [147]. In vitro experiments showed that Aβ oligomers inhibited HSV-1 infection, suggesting that Aβ accumulation may function as a defense mechanism against pathogens. A recent study showed that HSV-1 infection in the brains of transgenic 5xFAD mice accelerated AD progression by activating the NLRP3 inflammasome pathway [148]. Conversely, blocking NLRP3 inflammasome signaling reduced Aβ deposition and mitigated cognitive decline in 5xFAD mice following HSV-1 infection [148].

Despite these experimental insights, proving that AD originates from microbial infection remains challenging due to the long pre-symptomatic period and reliance on transgenic animal models. Supporting this, evidence from a cohort of 1002 dementia-free individuals aged 70 and residing in Sweden between 2001 and 2005 and followed for 15 years revealed that participants with anti-HSV IgG had more than twice the risk of developing dementia [149]. However, no statistically significant association was observed with AD, likely due to the low incidence of AD in the cohort, which may have limited the study’s statistical power [149]. Other herpes markers, including anti-HSV immunoglobulin M (IgM), anti-CMV IgG, and anti-herpesvirus treatment, showed no association with dementia or AD [149].

Recent studies suggest that Aβ may act as an antimicrobial peptide, aggregating in response to microbial infection [22]. While this response aims to combat pathogens, it may inadvertently contribute to AD pathogenesis by forming biofilm-like structures that harbor microbial communities. These biofilms, composed of microbial amyloids and human Aβ, create a persistent and complex environment in the brain [22]. For instance, HSV-1 infection in neuronal and glial cells leads to significant intracellular increases in Aβ1–40 and Aβ1–42 levels, accompanied by a reduction in APP levels [150]. Aβ1–42 deposits have also been detected in the brains of HSV-1-infected mice, indicating that HSV-1 can directly contribute to the formation of senile plaques, a hallmark of AD pathology [150]. Pathogens, particularly viral infections like HSV-1, modulate host cellular processes through virus–host PPIs, including those involved in Aβ aggregation and amyloid formation [32].

Thus, viral infections can indirectly promote amyloid-containing biofilm formation through the antimicrobial actions of Aβ, which aggregates in response to chronic viral or microbial infections. Additionally, these infections can directly enhance Aβ accumulation and biofilm development, as demonstrated by HSV-1’s ability to amplify both Aβ aggregation and biofilm formation. These findings highlight the complex interplay between viral infections, host defense mechanisms, and AD pathology.

### 5.3. Synergistic Viral Pathogenic Actions and AD

Systems bioinformatics analysis revealed that the reactivation of herpesviruses (HSV-1, HCMV, and EBV) during acute co-infection with SARS-CoV-2 could create a harmful interplay, amplifying neurodegenerative effects through synergistic pathogenic influences on AD-related processes [32]. These processes include responses to unfolded proteins, regulation of autophagy, oxidative stress responses, and Aβ formation [32]. Such interactions highlight the complex dynamics between multiple viral infections and their impact on AD development and/or progression.

Virus–virus interactions (VVIs) are common in nature and can significantly influence the outcome of an infection, resulting in either protective or immunopathological effects [151]. Co-infections can lead to synergistic effects, where viruses enhance each other’s pathogenicity, or antagonistic effects, where one virus suppresses the activity of another. These interactions can disrupt host cellular processes, increasing the risk of disease comorbidities, including NDs like AD.

Prior exposure to pathogens can alter immune responses to subsequent infections, mediated through immunological memory [152]. Similarly, vaccines and endogenous microbiota can also influence the outcome of a current viral infection via heterologous immunity, which can be beneficial or detrimental [153]. Therefore, VVIs and virus–microbiota interactions can significantly modulate the progression and severity of viral infections, contributing to the complex etiology of NDs like AD.

## 6. Cross-Kingdom Synergistic and Antagonistic Actions Between Viruses and Commensal Microbiota and Potential Implications for AD

Microorganisms within the human body do not exist in isolation but instead engage in intricate interactions with each other and the host, collectively shaping health and disease outcomes. The dynamic interplay between viruses and commensal microbiota forms a network of cross-kingdom interactions that can either exacerbate or mitigate disease processes. These relationships are particularly relevant in NDs like AD, where microbial communities profoundly influence immune responses, systemic inflammation, and neurodegeneration.

### 6.1. Role of Gut Microbiota in Modulating Viral Infections

Commensal microbiota play a critical role in shaping the outcomes of viral infections, either suppressing or enhancing viral replication through a variety of mechanisms [154,155]. For example, intestinal microbiota enhance infections with certain enteroviruses in mice, while antibiotic-treated mice exhibit reduced susceptibility to these infections and lower levels of viral replication in intestinal tissues [156,157,158,159,160]. However, the degree to which microbiota contribute to viral infectivity varies among enteroviruses; for instance, coxsackievirus depends more on microbiota for its infectivity compared to poliovirus [157]. While the gut microbiota are known to modulate enterovirus infections, the precise mechanisms underlying their role in enhancing viral pathogenesis remain unclear.

The gut microbiota are essential for the development and regulation of innate and adaptive immune responses, which are critical for protecting against viral infections [81]. By influencing these immune responses, microbiota can impact susceptibility to enteroviruses and other viral infections. Studies have reviewed the immunomodulatory effects of gut microbiota, highlighting the complex interactions between microbiota and the immune system in shaping responses to viral infections [161].

Metabolites produced by commensal bacteria can act as antimicrobial agents that directly target viruses, destabilizing virions or blocking viral receptors, thereby suppressing infection [162,163]. Conversely, some bacterial products may promote viral infections by enhancing virion stability or facilitating genetic recombination, which increases viral infectivity [164,165,166]. Commensal microbiota can indirectly influence viral infection outcomes by modulating both innate and adaptive immune system mechanisms, through the production of microbial metabolites such as SCFAs and NTs [167]. Depending on the context, these interactions can either enhance or suppress the host’s antiviral defenses.

The dynamic interplay between gut microbiota and viral infections suggests a significant role for microbiota in regulating viral pathogenesis. Dysbiosis or alterations in microbiota composition could influence viral infectivity, immune responses, and systemic inflammation, which are all linked to the progression of NDs like AD.

### 6.2. Viral Tropism and Gut–Brain Interactions in AD

Viruses display a wide range of tissue tropism, meaning they can specifically target and infect different types of tissues within the host. For example, HSV-1, strongly linked to AD, demonstrates a marked tropism for neural tissues. HSV-1 initially infects epithelial cells and then establishes latency in the peripheral nervous system, particularly in the trigeminal ganglia, and can periodically reactivate, spreading to the CNS [168]. Once in the brain, HSV-1 has been found in regions associated with AD pathology, such as the hippocampus and orbitofrontal cortex, potentially contributing to neurodegeneration [168].

HSV-1 and related neurotropic viruses, including HSV-2 and VZV, also establish latency in ganglionic neurons that innervate the GI tract [169,170,171,172,173]. These viruses initially infect mucosal epithelial cells, where they replicate during the lytic phase. Virions then spread to nearby cells and ganglionic neurons, establishing latency. Reactivation triggers the release of new virions, which migrate to the gut mucosa, initiating another lytic cycle. These neurotropic viruses also spread trans-synaptically, invading and replicating within the central, peripheral, and enteric nervous systems, highlighting their potential to disrupt gut–brain communication [169,170,171,172,173].

### 6.3. Disruption of Gut Microbiota and Immune Responses in Viral Infections

HSV-1 infection disrupts gut microbiota, triggering an antiviral immune response in microglia [174]. When gut microbiota are depleted through oral antibiotic treatment, microglial activation becomes excessively heightened, exacerbating herpes simplex encephalitis (HSE) pathology, a severe complication of HSV-1 infection [174]. This underscores the critical role of the gut microbiota in modulating immune responses and mitigating viral pathology.

Exogenous administration of nicotinamide N-oxide (NAMO), an oxidative derivative of nicotinamide primarily produced by intestinal neomycin-sensitive bacteria such as *Lactobacillus gasseri* and *Lactobacillus reuteri*, has been shown to significantly inhibit the progression of HSE. HSV-1 disrupts mitophagy and induces mitochondrial dysfunction, triggering microglial activation and proinflammatory cytokine production [174]. NAMO restores NAD+-dependent mitophagy, reducing microglial activation and preventing early HSV-1 infection in neuronal cells [174]. These findings suggest that the gut microbiota and their metabolites can influence viral infections and neuroinflammation, highlighting their potential role in AD pathogenesis.

### 6.4. Gut Dysbiosis and Viral-Associated Inflammation in AD

The interaction between viruses and gut microbiota may play a role in the onset and progression of AD. Although SARS-CoV-2 is an acute viral infection rather than a persistent one, it is associated with post-COVID-19 AD-like symptoms, such as memory impairment and brain fog [143,144,145]. During SARS-CoV-2 infection, gut dysbiosis is observed, characterized by an increase in opportunistic pathogens and a decline in beneficial commensal bacteria [175,176]. Importantly, this dysbiosis persists beyond the resolution of respiratory symptoms and after throat swabs test negative for SARS-CoV-2 [175,177]. Viral RNA has been detected in the feces of approximately 20% of patients after oral swabs test negative, indicating possible localized gastrointestinal involvement rather than prolonged infection [178].

Persistent gut dysbiosis during and after COVID-19 infection correlates with elevated plasma cytokine and chemokine levels, suggesting that alterations in microbiota composition may amplify immune responses and contribute to systemic inflammation [177]. Beneficial commensal bacteria, such as *Faecalibacterium prausnitzii*, *Eubacterium rectale*, and *Bifidobacteria*, known for their immunomodulatory effects, are significantly reduced during COVID-19 infection and remain depleted even after recovery [177]. This loss of microbial diversity may compromise immune regulation, exacerbate systemic inflammation, and potentially contribute to neuroinflammation and AD progression.

Although SARS-CoV-2 itself is not a persistent viral infection, its indirect effects on gut microbiota and systemic inflammation underscore the importance of viral-induced dysbiosis in AD pathogenesis. Understanding the interactions between transient viral infections, microbiota composition, and neuroinflammatory pathways may provide valuable insights into the mechanisms linking gut health and NDs.

## 7. Genetic Susceptibility: A Regulator of Microbial Composition and Viral Infections Outcome

### 7.1. Genetic Factors in Shaping the Microbiome and Influencing Disease Risk

Genetic susceptibility is a critical factor influencing an individual’s predisposition to complex diseases like AD and plays a significant role in determining microbial composition and the outcome of viral infections. The microbiome is shaped not only by environmental factors such as diet, lifestyle, antibiotic use, geography, medication use, and infection by pathogenic organisms, but also by genetic variability [154,179,180]. While some researchers argue that environmental perturbations outweigh genetic polymorphisms in shaping microbiome–host interactions [181], others find that host genetic susceptibility plays a stronger role in regulating microbiome composition [182].

Genetic variability can influence microbiota composition through multiple mechanisms. One possibility is that a genetic variant directly triggers a disease phenotype, with the resulting altered microbiome being a secondary effect of the disease state [182]. Alternatively, a genetic variant may alter gene expression in the host, which then changes the microbiome and leads to the disease phenotype [182]. Finally, a genetic variant can directly impact the microbiome, which subsequently causes the disease phenotype [182].

### 7.2. APOE Genotype and Gut Microbiome Composition

The APOE genotype is the most significant genetic risk factor for AD [183]. While many studies have explored its pathological mechanisms, the impact of the APOE genotype on gut microbiome composition remains understudied. Research has examined the relationship between the gut microbiome and APOE genotype in humans and APOE-targeted replacement (TR) transgenic mice. Sequencing the fecal microbiota of individuals with various APOE genotypes showed no notable differences in the overall diversity of microbial communities [184]. However, specific bacterial taxa showed differing relative abundance between the APOE-ε2, APOE-ε3, and APOE-ε4 genotypes, notably *Prevotellaceae* and *Ruminococcaceae*, as well as several butyrate-producing genera [184]. These findings were corroborated by gut microbiota comparisons in APOE-TR mice [184].

Another study indicated that the relative abundance of multiple bacterial taxa varied significantly depending on the APOE genotype, suggesting a robust link between APOE alleles and gut microbiome structure in this murine model [185]. Furthermore, it was shown that the gut microbiome in AD transgenic mice is significantly influenced by both APOE genotype and sex [186]. The relative abundance of specific bacterial genera such as *Prevotella* and *Ruminococcus* was higher in female APOE-ε4 mice compared to female APOE-ε3 mice, while *Sutterella* abundance was higher in male APOE-ε4 mice compared to male APOE-ε3 mice, indicating that the impact of APOE-ε4 on the gut microbiome is modulated by sex [186].

### 7.3. APOE-ε4 Allele and Viral Infection Susceptibility

The APOE-ε4 allele has a nuanced relationship with susceptibility to infections, offering protection against some viruses while increasing the risk for others [11,12]. For example, carriers of APOE-ε4 are more prone to infections like HSV-1 and EBV, often experiencing higher rates of infection or more severe disease outcomes [12]. In contrast, APOE-ε4 appears to provide a protective advantage against HCV and Hepatitis B Virus (HBV), lowering infection risk or enhancing immune regulation [12]. This dual role underscores the varied influence of APOE-ε4 on immune function, which differs depending on the specific viral pathogen.

### 7.4. Genetic Variability Drives Viral Susceptibility, Viral Evolution, and AD Pathogenesis

While viral infections are linked to AD, it is improbable that viruses alone can cause the disease, as viruses associated with AD are common in the population, yet AD prevalence is much lower [187]. Genetic susceptibility impacts how the immune system responds to pathogens, affecting the ability to clear acute infections or manage chronic ones [187,188]. An individual’s genetic makeup dictates the immune system’s specific sensitivity to different pathogens. This sensitivity does not imply an overall weakness in immune surveillance [187], but suggests that variability in each person’s “immunogenome” creates a heightened vulnerability to particular pathogens. This susceptibility can influence the symbiotic relationship between viruses and their hosts, leading to immune-related diseases through mechanisms such as modifying immune responses to particular viruses, changing the cell types that viruses can infect (viral tropism), and modifying the severity of viral infections (neurovirulence) [189,190,191].

Genetic variation within the human host exerts selective pressure on viruses, driving their evolution and affecting the dynamic relationship between viruses and the host [192]. Consequently, diversity in the genetic makeup of either the host or viral proteins can lead to alternations in PPIs between viruses and their hosts, resulting in the emergence of edgetic perturbations. These genetic variations can cause different responses to viral infections among individuals, influencing the course and severity of diseases, including AD. Understanding these interactions is crucial for elucidating how genetic variability affects viral pathogenicity and contributes to disease susceptibility [193], as well as for comprehending how it impacts the development of viral-associated diseases like AD. This knowledge can inform the development of targeted therapies and interventions that account for individual genetic differences, ultimately improving disease management and treatment outcomes.

## 8. Microbiome-Based Therapeutic Approaches for AD

### 8.1. Fecal and Oral Microbiome Transplantation

**Figure 2:**
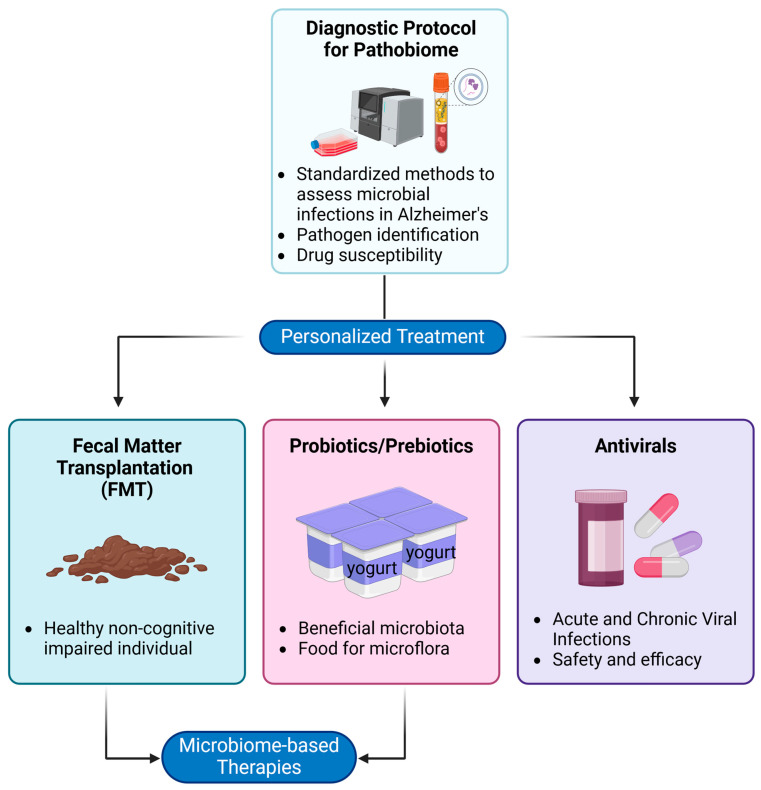
**Illustration of personalized treatment approaches based on microbiome-based and antiviral therapies for AD.** The diagnostic protocol for pathobiome involves standardized methods to assess microbial infections in AD, including pathogen identification and drug susceptibility testing. Based on these diagnostic results, personalized treatment strategies can be developed. These include FMT from a healthy, non-cognitively-impaired individual to restore a balanced gut microbiome, the use of probiotics and prebiotics to introduce beneficial microbiota and support microflora health, and antiviral treatments targeting acute and chronic viral infections with considerations for safety and efficacy. These strategies aim to leverage the benefits of microbiome modulation to mitigate AD pathology.

Recent research highlights the potential of microbiome-based therapies, such as FMT, in preventing and managing AD [194] (Figure 2). FMT, which involves transplanting fecal bacteria from a healthy donor to a patient, aims to restore gut microbiome balance. If FMT from patients with AD results in the initiation of AD in a different or the same organism [24], the reverse may also be true; restoring a healthy microbiome might mitigate AD pathology. For example, FMT has been shown to be effective in reducing Aβ plaques and improving cognitive function in mouse models of AD [38,39,40].

A compelling case report supports the potential of FMT in humans. An 82-year-old AD patient who underwent FMT for a *Clostridioides difficile* infection, using stool from his 85-year-old wife as the donor, exhibited significant improvement in cognitive symptoms two months post-FMT [195]. The evidence suggests that restoring a healthy gut microbiome through FMT might mitigate AD pathology. However, extensive clinical trials are needed to confirm these findings and standardize FMT as a therapeutic option.

Oral Microbiome Transplantation (OMT) is also being explored as a novel approach to restoring a healthy oral microbiome. This strategy could help to address oral dysbiosis linked to AD and other systemic diseases [196]. Future research aims to optimize OMT, identify “super-donors”, and validate its efficacy in AD and related conditions.

### 8.2. Probiotics and Prebiotics

Probiotics, which are live beneficial bacteria, and prebiotics, dietary fibers that nourish these bacteria, represent another avenue of therapy. Prebiotics and probiotics have demonstrated potential in reducing neuroinflammation, improving metabolic health, and enhancing cognitive function in AD models [197,198]. For instance, probiotics improved cognitive performance and reduced neurodegeneration in 3xTg-AD mice on a high-fat diet [199]. Several other preclinical and a few clinical studies have also demonstrated promising results of probiotics in improving cognitive function and reducing Aβ plaques, potentially mediated by their anti-inflammatory effects [200,201,202].

The potential benefits of probiotics in treating AD remain in the early stages of research, necessitating further studies to fully unravel the complex interactions between the host microbiome and AD pathology. While evidence suggests that AD patients exhibit distinct microbiota patterns compared to controls, findings on specific gut microbiome compositions are inconsistent across studies. For instance, one study reported an increased abundance of the phylum *Bacteroidetes* in predominantly Caucasian AD patients [203], while another study found decreased abundance of *Bacteroidetes* in Chinese AD patients compared to controls [204]. These discrepancies may arise from variations in patient demographics, lifestyle and dietary habits, or study methodologies. Gaining a deeper understanding of how these factors shape microbial composition is essential for developing tailored probiotic therapies.

Individual differences in microbiome composition, often influenced by “personalized microbiome determinants”, underscore the need for a customized approach to probiotic therapy. Future research should aim to identify the probiotic strains most beneficial for AD patients, optimize dosages and treatment durations, and determine the most effective delivery methods. Additionally, further investigation into the role of prebiotics in enhancing probiotic function and overall gut health could significantly bolster the therapeutic potential of these interventions.

### 8.3. Antiviral Treatments

The link between viral infections and AD has prompted investigations into antiviral therapies as potential treatment options. One clinical trial evaluated the efficacy of Apovir, a combination of two antiviral agents, pleconaril, targeting enteroviruses, and ribavirin, active against a range of viruses, to determine its potential in slowing AD progression [205]. The aim was to determine if this treatment could slow AD progression. However, the trial results were inconclusive due to a high dropout rate caused by the increased frequency and severity of adverse events linked to Apovir [205].

Despite these challenges, antiviral agents targeting HSV have shown promising results in preclinical studies. Animal models have demonstrated that these agents can effectively reduce Aβ accumulation, suggesting their potential in mitigating AD pathology [206,207]. Further research and clinical trials are needed to refine antiviral therapies, addressing safety concerns while exploring their role in AD treatment.

Evidence indicates that common infections diagnosed between ages 65 and 75, including shingles (caused by VZV), are significantly associated with an increased risk of AD later in life, with the risk increasing by 16% to 42% [208]. The live attenuated zoster vaccine, administered between the ages of 65 and 75, was associated with a 15–21% reduced risk of developing AD [208]. This protective effect was specific to AD, suggesting that vulnerability to VZV may play a distinct and significant role in its pathology compared to other dementias [208]. The observed benefits of vaccines underscore the importance of immunization strategies in the elderly population, not only for preventing infections but also for potentially reducing AD risk.

### 8.4. Targeting Biofilms and Dietary Interventions

Therapeutic strategies targeting biofilm formation in AD could offer another approach. Agents that disrupt biofilms or enhance antibiotic penetration may mitigate their role in AD progression. Additionally, dietary interventions promoting a healthy microbiome could reduce biofilm formation and improve overall gut health. Integrating these strategies with existing therapies may provide a comprehensive framework to address the microbial and pathological factors contributing to AD.

Further research is necessary to refine these microbiome-based therapies, ensuring their efficacy and safety in clinical applications for AD.

## 9. Conclusions

Recent advancements in AD research underscore the potential role of microbial infections in AD pathogenesis. Evidence increasingly suggests that microbial communities, including the gut microbiome, brain biofilms, and oral microbiome, may contribute to the development and progression of AD. Acute and chronic viral infections, such as those caused by EBV, HSV-1, and SARS-CoV-2, are also strongly associated with an elevated risk of developing AD. However, further research is essential to elucidate the precise mechanisms through which these microbial factors influence AD susceptibility and progression. Such studies are critical for advancing targeted interventions to mitigate microbial contributions to AD and improve care for those at risk of or suffering from the disease.

Microorganisms should not be studied in isolation, as their interactions with each other and with the host play a critical role in AD pathogenesis. For instance, gut and oral microbiota, brain biofilms, and viruses often interact synergistically or antagonistically, amplifying or mitigating their individual effects. These cross-kingdom interactions, including competition, biofilm formation, and immune modulation, highlight the complexity of microbial contributions to AD. Understanding these dynamic interactions is crucial for developing holistic therapeutic approaches that target not only individual pathogens, but also the microbial ecosystem as a whole.

This review aimed to address the significant gaps in understanding the role of microbial infections in AD pathogenesis by proposing several testable hypotheses. These hypotheses focus on clarifying the mechanisms through which gut microbiota, brain biofilms, oral microbiomes, and viral infections contribute to AD development and progression. Specific research directions include: investigating the causal relationship between gut microbiota dysbiosis and AD by analyzing changes in microbial populations and their metabolites in AD patients versus healthy controls; exploring the potential for gut-derived Aβ to reach the brain via the bloodstream and vagus nerve, as well as its subsequent role in amyloid plaque formation; assessing the impact of microbial amyloids, such as curli produced by *Escherichia coli* and *Pseudomonas aeruginosa*, on Aβ aggregation in the brain; evaluating the role of oral microbiome dysbiosis and periodontal disease in promoting neuroinflammation and AD pathology; and examining the influence of viral infections, particularly EBV, HSV-1, and SARS-CoV-2, on AD pathogenesis through mechanisms such as viral-induced neuroinflammation and direct modulation of Aβ production.

By addressing these hypotheses, future research can provide critical insights into the complex interplay between microbial communities and host processes in AD, ultimately contributing to the development of targeted microbiome-based therapies and interventions.

In conclusion, this study emphasizes the fundamental role of microbial agents, particularly microbiota and viruses, in AD pathogenesis, and how they directly or indirectly contribute to the emergence of pathological mechanisms associated with the disease. These mechanisms include Aβ accumulation, chronic neuroinflammation, dysregulated lipid metabolism, increased BBB permeability, and disruptions in neurotransmitter synthesis. Understanding these intricate interactions is essential for uncovering how microbial agents influence disease onset and progression, paving the way for novel therapeutic strategies.

## 10. Current Challenges and Future Research Directions

While promising, the field faces several challenges in studying microbial pathogenesis in AD. Variability in findings often arises from differences in study design, populations, and methodologies. To address these limitations, there is a growing emphasis on establishing standardized methodologies to assess microbial infections in patients exhibiting early signs of AD, such as mild cognitive impairment (MCI), or those at risk of AD [209]. These standardized methodologies include uniform sampling techniques, consistent protocols for DNA/RNA extraction, advanced sequencing technologies for comprehensive microbial profiling, and the development of rapid and accurate diagnostic assays. Stringent and reproducible tests are required to provide evidence-based proof of the involvement of specific microbes in AD pathoetiology. These tests should not only demonstrate the involvement of microbial organisms in the clinical deficits associated with AD but also suggest appropriate antimicrobial modulators to mitigate these deficits.

The Alzheimer’s Pathobiome Initiative recently proposed the development of a consensus diagnostic protocol as a critical step forward in investigating the brain pathobiome in patients with MCI and AD [209]. This protocol aims to standardize procedures for sampling, identifying, and analyzing microbial communities within the brain and other relevant areas, ensuring consistency and reproducibility across studies [209].

In addition to standardization efforts, understanding the dual roles of Aβ, including its pathological aggregation and potential antimicrobial functions, is essential for developing balanced therapeutic approaches. Future research should investigate the potential beneficial physiological antimicrobial roles of Aβ and determine whether only Aβ plaques should be targeted for AD treatment rather than Aβ peptides [99]. This approach ensures that the beneficial effects of Aβ peptide are preserved while effectively addressing its pathological roles in AD, including that of the formation of Aβ plaques [99].

When designing therapeutic approaches aimed at restoring microbiota homeostasis, it is also important to understand the synergistic and antagonistic effects between microbiota and viral infections. A recent study has shown that EBV, which establishes chronic infection within the human host, can modulate immune system processes via virus–host PPIs, similarly to how microbiota, specifically from the *Lactobacillus* and *Bacillus* genera, modulate these processes through their metabolic products [52]. *Lactobacillus* is known as a “friendly” bacterium, living symbiotically within the host and promoting health by forming biofilms that block the adhesion of pathogenic microbes, hence defending against harmful pathogens [210]. Additionally, *Lactobacillus* and *Bacillus* genera are common in supplements containing probiotics, with *Lactobacillus* being the most frequently used genus [211,212]. Understanding these interactions is essential for developing effective microbiome-based therapies for AD that leverage the health-promoting effects of beneficial bacteria while mitigating the impacts of viral infections.

Various factors, including a weakened immune system, diet, viral infections, and genetic predispositions, contribute to microbiome changes in diseases like AD. However, understanding how these factors shape disease-specific outcomes is crucial. For instance, FMT from AD patients into rodents induces cognitive impairments linked to hippocampal neurogenesis [24], while FMT from Parkinson’s disease (PD) patients into mice induces PD-like symptoms [213], emphasizing the distinct role of microbiota in disease phenotypes. Although dysbiosis is common in NDs, specific microbial shifts critically influence pathology. Future research should investigate cross-condition FMT experiments, such as introducing FMT from PD patients into AD models and vice versa, to determine whether microbiota changes are condition-specific or driven by shared pathways. Such studies could reveal key microbiota-driven mechanisms and support the development of targeted microbiome-based therapies for NDs.

To advance this field, future research should focus on clarifying the causal relationships between microbial dysbiosis and AD, focusing on microbial metabolites, amyloidogenic proteins, and host immune responses. For example, elucidating how microbial amyloids exacerbate amyloid-beta aggregation or how gut-derived metabolites modulate microglial activity could provide insights into the initiation of neurodegenerative cascades.

Additionally, identifying specific microbial signatures or virome alterations associated with early-stage AD may enable the development of predictive biomarkers and personalized treatment approaches. The integration of microbiome-focused interventions, such as probiotics, prebiotics, FMT, and antiviral therapies, holds promise for mitigating microbial contributions to AD. Moreover, exploring dietary and lifestyle modifications that support microbiome health could complement pharmacological therapies, offering a holistic approach to disease management.
